# Integrative Network-based Analysis of Magnetic Resonance Spectroscopy and Genome Wide Expression in Glioblastoma multiforme

**DOI:** 10.1038/srep29052

**Published:** 2016-06-28

**Authors:** Dieter Henrik Heiland, Irina Mader, Pascal Schlosser, Dietmar Pfeifer, Maria Stella Carro, Thomas Lange, Ralf Schwarzwald, Ioannis Vasilikos, Horst Urbach, Astrid Weyerbrock

**Affiliations:** 1 Department of Neurosurgery, Medical Center University of Freiburg, Freiburg, Germany; 2 Department of Neuroradiology, Medical Center University of Freiburg, Freiburg, Germany; 3 Institute for Medical Biometry and Statistics, Medical Center University of Freiburg, Freiburg, Germany; 4 Department of Hematology, Oncology and Stem Cell Transplantation, Medical Center University of Freiburg, Freiburg, Germany; 5 Department of Medical Physics, Diagnostic Radiology, Medical Center University of Freiburg, Freiburg, Germany

## Abstract

The goal of this study was to identify correlations between metabolites from proton MR spectroscopy and genetic pathway activity in glioblastoma multiforme (GBM). Twenty patients with primary GBM were analysed by short echo-time chemical shift imaging and genome-wide expression analyses. Weighed Gene Co-Expression Analysis was used for an integrative analysis of imaging and genetic data. N-acetylaspartate, normalised to the contralateral healthy side (nNAA), was significantly correlated to oligodendrocytic and neural development. For normalised creatine (nCr), a group with low nCr was linked to the mesenchymal subtype, while high nCr could be assigned to the proneural subtype. Moreover, clustering of normalised glutamine and glutamate (nGlx) revealed two groups, one with high nGlx being attributed to the neural subtype, and one with low nGlx associated with the classical subtype. Hence, the metabolites nNAA, nCr, and nGlx correlate with a specific gene expression pattern reflecting the previously described subtypes of GBM. Moreover high nNAA was associated with better clinical prognosis, whereas patients with lower nNAA revealed a shorter progression-free survival (PFS).

Glioblastoma multiforme (GBM) is the most common primary malignant brain tumour in adults, with an incidence of 3–4 cases per 100,000 people^1^. In spite of the best available treatment, the prognosis for patients with recurrent GBM is poor, with a median survival of not more than 25–40 weeks^2^,^3^,^4^,^5^,^6^.

Proton MR Spectroscopy (^1^H MRS) of brain tumours measures a variety of metabolites being attributed to different biological functions, *e.g.* N-acetylaspartate (NAA) to healthy neurons^7^ or invasion of tumour^8^, creatine (Cr) to energy metabolism^9^, choline to cell membrane metabolism^10^,^11^, glutamine and glutamate (Glx) to anaerobic metabolism of cancer cells^12^,^13^ and intracellular neurotransmitters^14^, and myo-inositol (Ino) to glial proliferation^15^ (for a review see Callot *et al*.^16^ and the literature cited in there)^16^. Diehn *et al*.^17^ described different radiogenomic MR imaging traits corresponding to specific gene expression patterns. For example, hypoxia-related tumours had a strong contrast enhancement and correlated with genes belonging to a hypoxia gene set, such as *VEGF*, *Serpine, ADM* or *PLAUR*. Tumours with severe mass effect had an increased expression of genes associated with proliferation. Two subtypes were identified to have a specific clinical impact: patients with an infiltrative imaging pattern had a worse outcome in comparison to patients with an oedematous appearance. Between both groups, typically mesenchymal or proneural genes (OLIG1, OLIG2, SOX6) were differently expressed^17^. Furthermore, an enrichment of gene sets of CNS development and developmental functions were described in the infiltrative subtype. These findings suggest a higher fraction of stem cells in the infiltrative subgroup^17^. Another study used the same radiogenomic types as described by Diehn *et al*.^17^ for an extended molecular analysis, and added expression datasets and copy number variants to find specific pathway correlations^17^. LTBP1 and RUNX3 were identified in the contrast-enhancing subtype and CHI3L1 was significantly higher expressed in a subgroup associated with subventricular zone involvement^18^. A recent study by Bourdillion *et al*. used ^1^H MRS analysis to identify a metabolic pattern that correlated to malignant transformation in low-grade tumours^19^. The Cho/Cr-ratio was found to be a powerful tool to monitor anaplastic transformation in low-grade tumours. Pope *et al*.^20^ published a study with 52 malignant gliomas with incompletely (IE) or completely contrast enhancing tumour (CE)^20^. Genetic analysis with microarray data showed an enrichment of proneural markers as OLIG2 in the IE tumours, whereas CE tumours expressed VEGF, MMP7 and Matrix Gla proteins. Zinn *et al*.^21^ analysed 78 patients of the TCGA database to identify genes being differentially expressed depending on the volume of FLAIR signal hyperintensity in MR^21^. POSTN was identified as to be differentially expressed. It was also enriched in the mesenchymal subgroup and significantly associated with a worse clinical outcome. Two recently published studies showed a connection between hyperperfusion of GBM, EGFR expression and *EGFRvIII* mutation^22^,^23^. Gevaert *et al*.^24^ analysed TCGA data and found genes of the mesenchymal subgroup being enriched in samples with a radiogenotypic feature called “oedema minimum histogram intensity”^24^.

So far, no spectroscopic data have been included into gene expression analyses of glioblastoma multiforme. Thus, the purpose of this study was to evaluate metabolites from ^1^H-MRS and to identify specific metabolic and genetic profiles of prospectively sampled tumours. A connection between different metabolic profiles and specific pathway activation or deactivation of glioblastoma multiforme has been sought for by an integrative analysis of genetic and spectroscopic data.

## Results

**Figure 1:**
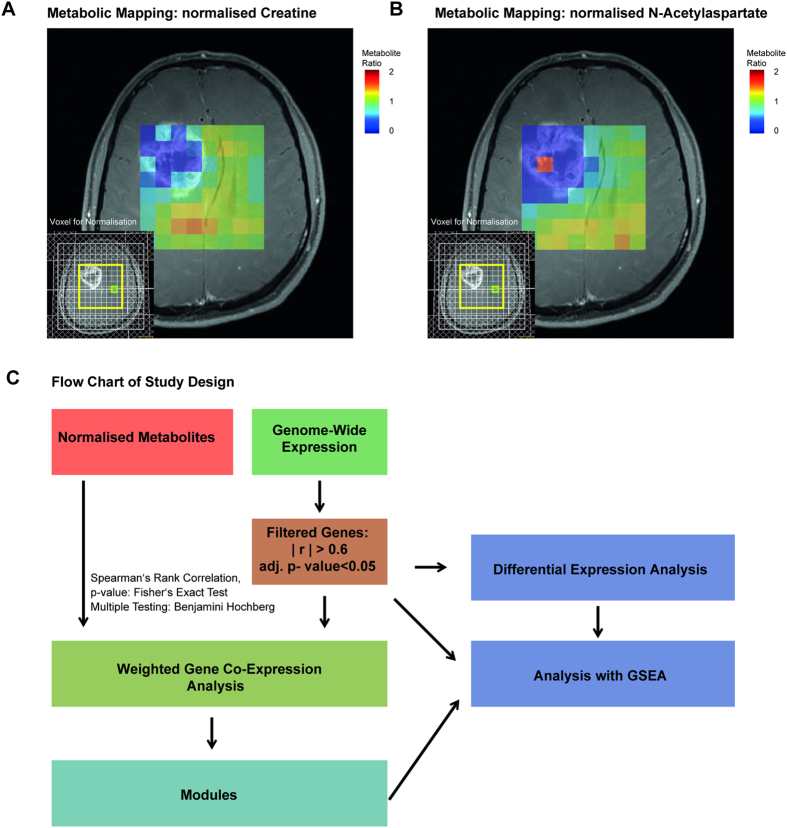
(**A,B**) Metabolite maps of nCr and nNAA of a 42-year old patient (BT_447) with a GBM in the right frontal lobe. Metabolite maps were overlaid on a contrast-enhanced T1-weighted image. The volume of interest (PRESS volume) is indicated in yellow, the reference voxel for metabolite concentration normalisation in green. It has to be noted that the high nNAA concentration in one voxel in the centre of the tumour is due to erroneous peak assignment by *LC*Model. (**C**) Flow chart of study design and data preparation.

**Figure 2:**
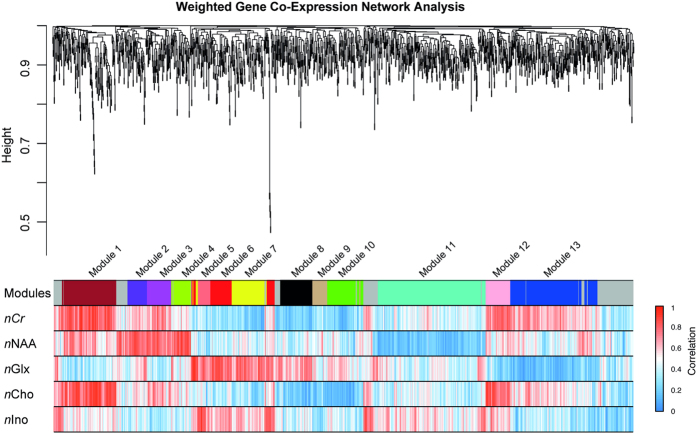
Weighted Gene Co-Expression Analysis of significantly (FDR < 0.05) metabolite-correlated genes (n = 1600). A soft threshold approach was used with a power of 18 (based on Scales Free Topology, SFT) in a signed network with dynamic branch cutting (deep split = 2). WGCNA identified 13 different modules. Bars below the modules show the direct correlation of each metabolite and corresponding genes. High correlation values are indicated by red, negative correlations by blue colour.

A workflow of the bioinformatical analysis and examples of metabolic maps are displayed in Fig. 1. WGCNA identified 13 different modules as shown in Fig. 2. These modules contained highly connected genes that represent certain biological functions. Intramodule connectivity (kME) was calculated and correlated to each module to identify highly correlated modules of each metabolite.

### N-Acetylaspartate

**Figure 3:**
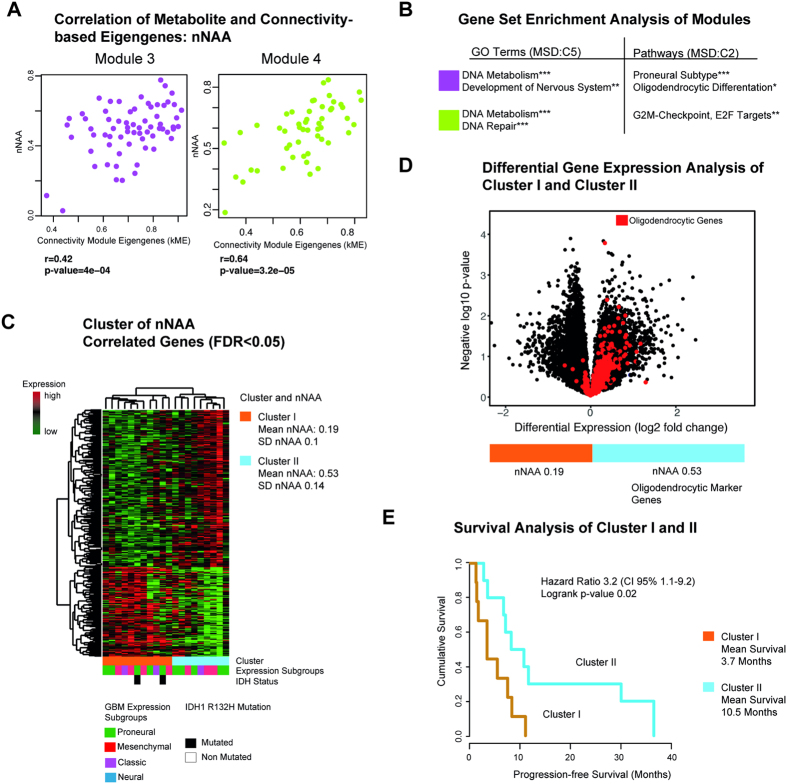
(**A**) Correlation of nNAA and connectivity-based module eigengenes of modules three and four. (**B**) GSEA identifies biological functions and GO Terms (MSD v5.1 C5), and associated pathways (MSD v5.1 C2, H1). (**C**) nNAA-associated genes are clustered by Spearman’s rank correlation into two clusters. Bars below the heatmap describe the IDH1-status and the expression subgroup of each patient. (**D**) Volcano plot of differentially expressed genes of cluster I and II. Genes of the oligodendrocytic gene set^25^ are marked in red. (**E**) Kaplan Meier curves of progression-free survival for clusters I and II. Significance level of the corrected p-values is indicated as *p < 0.05, **p < 0.01, ***p < 0.001.

Two modules correlating with nNAA were found. The intramodule connectivity based on eigengene correlation (kME) of module 3 was positively correlated with nNAA (r = 0.42, p < 0.01), and associated with DNA metabolism, development of the nervous system and oligodendrocytic differentiation as found by GSEA, Fig. 3A,B. Module 4 (r = 0.64, p < 0.01) was associated with DNA metabolism and DNA repair mechanism. In module 4, G2M checkpoints and genes of the E2F targets were significantly enriched. Hierarchical clustering of the genes correlated with nNAA identified two groups, (Fig. 3C). Cluster I had a mean nNAA of 0.2 ± 0.09 [dimensionless ratio], cluster II showed a higher nNAA with a mean of 0.51 ± 0.16. Both clusters were tested by differential gene expression analysis (Fig. 3C). In the cluster with high nNAA, a significant enrichment of genes was found belonging to oligodendrocytic and neural development as described by Cahoy *et al*.^25^, (Fig. 3D). In the cluster group with low nNAA, several marker genes, amongst others, of astrocytic and glial development could be identified.

### Creatine and Choline

**Figure 4:**
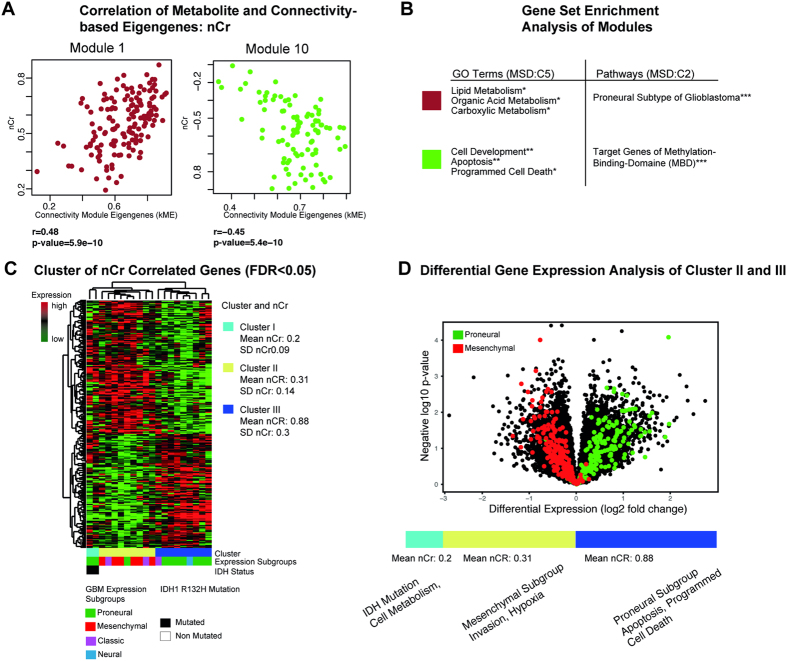
(**A**) Correlation of nCr and connectivity-based module eigengenes of modules one and ten. (**B**) GSEA identifies biological functions and GO Terms (MSD v5.1 C5), and associated pathways (MSD v5.1 C2, H1). (**C**) nCr associated genes are clustered by Spearman’s rank correlation into three clusters. Bars below the heatmap describe the IDH1-status and the expression subgroup of each patient. (**D**) Volcano plot of differential gene expression analysis. Genes of the mesenchymal subgroup are marked in red, proneural genes in green. The significance level of the corrected p-values is indicated as *p < 0.05, **p < 0.01, ***p < 0.001.

Two modules with a significant correlation between module kME and nCr values were identified (Modules 1, 10). Analysis of connectivity-based eigengenes revealed two significant modules being correlated with nCr (module 1: r = 0.48, p < 0.01; module 10: r < −0.45, p < 0.01), Fig. 4A. GSEA showed significantly enriched gene sets for each module, Fig. 4B. In module 1, a strong enrichment of genes was found belonging to the proneural expression subtype, described by Verhaak *et al*.^26^. In addition, a high association of module 1 to lipid and acid metabolism was detected. Module 10 was associated with cell death and apoptosis. A highly significant enrichment was present for genes that belong to the Methyl-binding-Domain (MBD). A cluster analysis of nCr-correlated genes showed 3 different groups, Fig. 4C. Cluster I with extremely low nCr (mean nCr 0.2 ± 0.09, dimensionless ratio) was associated with IDH1 mutation status and corresponded to the proneural subtype. Cluster II (mean nCr of 0.34 ± 0.1) was associated with samples of the mesenchymal and classical subtype. In cluster III (mean nCr of 0.83 ± 0.3), GBM belonging to the proneural and neural subtype were represented. A GSEA between cluster II and III confirmed a highly significant enrichment for mesenchymal genes in cluster II, and proneural marker genes in cluster III, Fig. 4D. No significant differences of survival could be found between the three clusters defined by nCr.

For nCho and nCr similar correlations with the eigengenes of modules 1, 5, 7, 8, 10, 12 were found, Fig. 2. By analysing the kME of nCho, however, only module 12 was identified being negatively correlated to nCho. In module 12, further characterisation by GSEA was not successful.

### Glutamine and Glutamate and Inositol

**Figure 5:**
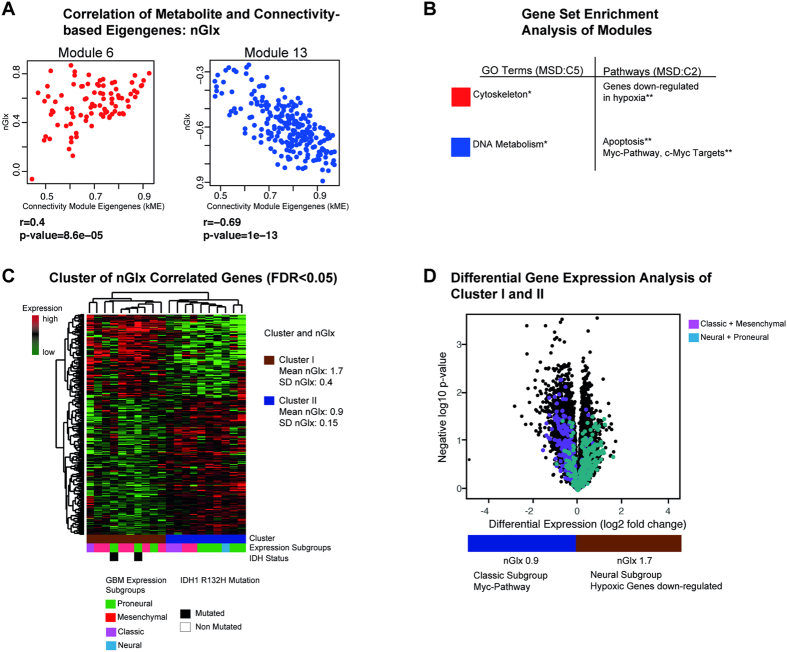
(**A**) Correlation of nGlx and connectivity-based module eigengenes of Module six and 13. (**B**) GSEA identifies biological functions and GO Terms (MSD v5.1 C5), and associated pathways (MSD v5.1 C2, H1). (**C**) nGlx-associated genes are clustered by Spearman’s rank correlation into two clusters. Bars below the heatmap describe IDH1-status and expression subgroup of each patient. (**D**) Volcano plot of differential gene expression analysis. Genes of the neural subgroup are marked in cyan, those of the classic subgroup in purple. The significance level of the corrected p-values is indicated as *p < 0.05, **p < 0.01, ***p < 0.001.

nGlx was significantly correlated with module 6 (r = 0.4, p < 0.01). In this module, GSEA identified genes being down-regulated in hypoxia and functionally involved in cytoskeletal processes. Module 13 was negatively correlated with nGlx (r = −0.69, p < 0.01), Fig. 5A. GSEA found genes of module 13 as to be involved in DNA metabolism and apoptosis, Fig. 5B. A cluster analysis of nGlx-correlated genes revealed two clusters: cluster I showed a mean nGlx of 1.7 ± 0.4 (dimensionless ratio), cluster II one of 0.9 ± 0.15, Fig. 5C. In cluster I, a GSEA showed a significant enrichment of marker genes belonging to the neural subgroup. In cluster II, significantly enriched genes of the classical subtype were present, Fig. 5D.

For nIno no significant correlations with kME of any module was found.

### Survival Analysis

The metabolite nNAA was associated with a better clinical outcome. Progression-free survival (PFS) revealed a mean of 3.7 months (CI 95% 1.9–6.9 months) in the group with low nNAA, and a mean of 10.5 months (CI 95% 5.1–21.9 months) in the group with high nNAA, Fig. 3E. This difference was significant in the log-ranked test (p = 0.02). In the Cox regression model, the calculated hazard ratio (HR) was 3.4 (CI 95% 1.1–9.2). Other metabolites did not show any differences in the survival analysis.

## Discussion

The quantified and normalised metabolites from spectroscopy were in line with the literature for contrast-enhancing tumour and contralateral normal appearing matter^27^. The metabolite NAA is deeply involved in oligodendrocytic development as described by Nordengen *et al*.^28^ and Baslow *et al*.^29^. The finding of a highly significant enrichment of genes associated with high nNAA supporting oligodendrocytic development and differentiation is corroborated by these publications. As shown in the volcanoplot (Fig. 3D), oligodendrocytic marker genes were stronger expressed in tumours with high nNAA than in those with low nNAA^25^. For progression-free survival (PFS), only patients with low or high nNAA showed any significant difference in this study. Patients clustered in the high nNAA group had significant longer PFS, Fig. 3E. Overall survival data is still pending, and was not integrated in this analysis. The finding of different PFS, however, appears to be independent of the IDH1 mutation, as both IDH1 mutated patients are contained in the low nNAA cluster. To exclude any influence of the IDH1 mutation on PFS, both IDH1 mutant patients were removed, and still a significantly different PFS between both clusters could be seen.

Creatine was identified as an important metabolite for a molecular characterisation of glioblastomas. Three clusters were associated with subgroups of distinct clinical courses^30^. Extremely low nCr was found in the presence of an IDH1 mutation. In this case, the citrate cycle is defective, and the tumour cells need an alternative supply of energy-rich derivates. Although phosphorylated and non-phosphorylated Cr cannot be distinguished by proton spectroscopy, this strong reduction of nCr points to a severe disturbance of the energy metabolism of the tumour, possibly resulting in a severe decrease of intracellular Cr. Activation of the hypoxia pathway, including *HIF1α*, is an important factor for a high invasiveness of mesenchymal tumours^31^. In the analysis of IDH wild type, samples with genes related to a low level of nCr belonged to signature genes of the hypoxic and mesenchymal subtype, whereas proneural target genes and up-regulated marker genes of apoptosis and programmed cell death were correlated with high nCr. This might be due to higher energy consumption in the mesenchymal subgroup. These findings are new, and cannot be compared to the literature. As mentioned above, the normalised values of Cr in contrast-enhancing tumour to the contralateral side were in line with the literature^27^. Since the Cr concentration has been shown to be relatively stable in many brain pathologies, Cr is often used as a reference metabolite in quantitative MR spectroscopy when reporting metabolite ratios. However, for MR spectroscopy studies in oncology the value of Cr seems to have been underestimated so far.

Glutamine is an amino acid and the storage form of the excitatory neurotransmitter glutamate in the central nervous system. Moreover, glutamine is one of the most important alternative energy derivates in tumours^12^. In this study, nGlx was negatively correlated with the myc-pathway and apoptotic functions. Myc-regulated genes seem to need glutamine as an energetic substrate for their metabolism^12^,^32^. An enrichment of neural marker genes in tumours with high nGlx could be found, leading to the hypothesis that the neural subtype could be reflected by high nGlx levels. On the other hand, low nGlx levels seem to belong to the classical/mesenchymal subgroup that is more invasive and has a worse prognosis^30^.

Our study has strong limitations because of the small number of cases and the incomplete overall survival data. Conservative statistical methods with corrections for multiple testing at each level of analysis were applied. Only corrected p-values (Bonferroni, Benjamini-Hochberg) were reported for the sake of robustness. Nevertheless, these findings have to be confirmed in a larger cohort of patients. Another critical factor is the selection of spectra. As mentioned above, in case of several spectra with contrast-enhancing tumour, the spectrum with the smallest full width at half maximum (FWHM) in *LC*Model was used. So the spectrum with the best quality was chosen, and a subjective bias was excluded. The issue of 2-hydroxyglutarate (2HG) detection arises for the two cases with IDH-mutation. Although 2HG was contained in the spectral basis for *LC*Model quantification, this metabolite could not be detected in any of the spectra. Another metabolite being not used in further analysis is lactate. At short echo-time spectroscopy it is overlaid by strong lipid resonances in GBM. This complicates detection and quantification and reduces the informative value of this metabolite.

This study realises radiogenomic mapping of glioblastoma multiforme by proton MR spectroscopy and genome-wide expression profiling. Patients in the cluster with high nNAA have a longer progression-free interval than those with low nNAA (Table 1). nCr and nGlx seem to distinguish between different expression subgroups of GBM as previously described by Verhaak *et al*.^26^ (Table 1). Low nCr tumours have an increased expression of hypoxia genes and enriched mesenchymal marker genes, while tumours with high nCr show a proneural subtype signature. Tumours with a high nGlx have enriched genes of the neural/proneural subtype, those with low nGlx an enrichment of the classical/mesenchymal signature.

## Methods

### Patients

For this prospective study we included 23 patients (median age 66 years, range 41–84 years), who underwent surgery at the department of neurosurgery between 2012 and 2014. The local ethics committee of the University of Freiburg approved data evaluation, imaging procedures and experimental design (protocol 100020/09 and 5565/15). The methods were carried out in accordance with the approved guidelines. Written informed consent was obtained from all patients.

Inclusion criteria were: (1) age older than 18 years, (2) preoperative MRI with H-MRS, (3) intraoperative MRI-guided sampling of tumour tissue from contrast-enhancing tumour, (4) histopathological confirmation of a glioblastoma multiforme (WHO criteria) (5) three and six-months follow-up with contrast-enhanced MRI. From those 23 patients, 20 patients could be enrolled into this study (Supplementary Table 1), 3 patients had insufficient follow-up. Progression-free survival (PFS) and overall survival (OS) was available for 18 patients. Two patients had no tumour progression until the end of 2015, and 7 patients had outstanding overall survival (OS) and are still alive. The Kaplan-Meier method was used to provide median point estimates and time-specific rates. The Hazard-Ratio (HR) was calculated by Cox-Regressions tests.

### Tissue collection and histology

Tumour tissue was sampled from contrast enhancing regions identified by intraoperative neuronavigation (Cranial Map Neuronavigation Cart 2, Stryker, Freiburg, Germany) during tumour resection. The tissue was snap-frozen in liquid nitrogen immediately and processed for further genetic analysis. Tissue samples were fixed using 4% phosphate buffered formaldehyde and paraffin-embedded with standard procedures. H&E staining was performed on 4 μm paraffin sections using standard protocols. Immunohistochemistry was applied using an autostainer (Dako) after heat-induced epitope retrieval in citrate buffer. IDH1 mutation was assessed by immunohistochemistry using an anti-IDH1-R123 antibody (1:20, Dianova).

### MR imaging and spectroscopy

MR imaging was performed on a 3T system (Magnetom TIM TRIO, Siemens, Erlangen, Germany) using a 12-channel head coil for signal reception. The imaging protocol for pre-operative and follow-up imaging included a 3D T2-weighted fluid-attenuated sequence, and a 3D T1-weighted magnetization prepared rapid gradient echo sequence before and after contrast application. For 2D chemical shift imaging (CSI), a point resolved spectroscopy (PRESS) sequence with outer volume saturation was used (TR: 1500 ms; TE: 30 ms; voxel size: 10 × 10 × 15 mm^3^). The volume of interest was placed so that the bone of the skull was not included.

### Post-processing and evaluation

Spectra were evaluated by using *LC*Model^33^. N-acetylaspartate and N-acetylaspartylglutamate were summarised as “NAA”, the choline-containing compounds glycerophosphocholine and phosphocholine as “Cho”, glutamate and glutamine as “Glx”. Creatine (Cr) and myo-inositol (Ino) were also fitted. A normalisation to the contralateral normal appearing matter was performed. Therefore, all metabolites were named with an “n” for “normalised” such as nIno = Ino_ipsi_/Ino_contra_, nCho = Cho_ipsi_/Cho_contra_, nCr = Cr_ispi_/Cr_contra_, nGlx = Glx_ipsi_/Glx_contra_, and nNAA = NAA_ipsi_/NAA_contra_. Voxels with contrast enhancing tumour and contralateral normal appearing matter (for normalisation to the contralateral side) were selected. In case of several available spectra, the one with the smallest line width and best baseline was chosen. The mean FWHM was 0.068 ppm (range 0.038–0.143 ppm). Examples of metabolite maps are given in Fig. 1.

### Genome-Wide Expression Analysis

RNA was prepared using the RNAeasy kit (Qiagen). An amount of 1.5 μg RNA was obtained for expression arrays analysis. Arrays were performed by human genome 2.0 chip (Affymetrix). Raw data were processed, normalized and controlled by R software and the Affymetrix R-package. Different expression analysis and statistical testing (pairwise t-test) were performed by limma R-package.

### Data Preparation and Analysis of MRS based Correlation

First, a Spermann’s rank correlation was performed to separate genes that correlate with each metabolite (|r| < 0.6). Fischer’s exact test was applied to separate genes by its p-value (p < 0.05) adjusted for multiple testing (Benjamini Hochberg^34^ as false discovery rate, FDR). Only genes being significantly correlated to a metabolite (FDR p-value < 0.05) were used as input for Weighted Gene Co-Expression Network Analysis (WGCNA).

### Weighted Gene Co-Expression Network Analysis and Gene Set Enrichment Analysis

WGCNA uses the topological overlapping measurement to identify corresponding modules as shown in Fig. 2. These modules were analysed by their eigengene correlation to each metabolite. The WGCNA analysis is a robust tool for integrative network analysis and was used in several recent studies^35^,^36^,^37^. In addition, a permutation-based pre-ranked Gene Set Enrichment Analysis (GSEA) was applied to each module to verify its biological functions and pathways^38^. The predefined gene sets of the Molecular Signature Database v5.1 were taken. For significant enrichment, p-values were adjusted by FDR. For a detailed description and R-code see the Supplementary information S1.

## Additional Information

**How to cite this article**: Heiland, D. H. *et al*. Integrative Network-based Analysis of Magnetic Resonance Spectroscopy and Genome Wide Expression in Glioblastoma multiforme. *Sci. Rep.* **6**, 29052; doi: 10.1038/srep29052 (2016).

## Supplementary Material

Supplementary Information

Supplementary Information

## Figures and Tables

**Table 1:** Summarised metabolic ratios in different subgroups.

	**nNAA (Dimensionless Ratio)**
Short Survivor	0.2 ± 0.09
Long Survivor	0.51 ± 0.16
	**nCR (Dimensionless Ratio)**
IDH mutation	0.2 ± 0.09
IDH wt	0.67 ± 0.3
IDHwt Proneural	0.83 ± 0.3
IDHwt Mesenchymal	0.34 ± 0.1
	**nGLX (Dimensionless Ratio)**
Classic/Mesenchymal	0.9 ± 0.15
Neural/Proneural	1.7 ± 0.4
