# Supplementary material for: Integrative Network-based Analysis of Magnetic Resonance Spectroscopy and Genome Wide Expression in Glioblastoma multiforme
Source: Sci Rep. 2016 Jun 28;6:29052. doi: 10.1038/srep29052 (PMC4924099; doi:10.1038/srep29052)
Supplement: Supplementary Information [file srep29052-s1.pdf]

Supplementary: Methods

Integrative Network-based Analysis of Magnetic Resonance Spectroscopy and Genome Wide Expression in Glioblastoma multiforme

Dieter Henrik Heiland[1]†*, Irina Mader[2]†, Pascal Schlosser[3], Dietmar Pfeifer[4], Maria Stella Carro[1] Thomas Lange[5], Ralf Schwarzwald[2], Ioannis Vasilikos[1], Horst Urbach[2], Astrid Weyerbrock [1]

[1]Department of Neurosurgery, [2]Department of Neuroradiology, [3]Institute for Medical Biometry and Statistics, [4]Department of Hematology, Oncology and Stem Cell Transplantation, [5]Department of Medical Physics, Diagnostic Radiology, Medical Center University of Freiburg, Freiburg, Germany

Corresponding author:
Dieter Henrik Heiland
Department of Neurosurgery
Medical Center University of Freiburg
Breisacher Strasse 64
79106 Freiburg
-Germany-
Tel: +49 (0) 761 270 50010
Fax: +49 (0) 761 270 51020
E-mail: dieter.henrik.heiland@uniklinik-freiburg.de

Methods

Weighted Gene Co-Expression Network Analysis (WGCNA)

The WGCNA is a tool first described by Steve Horvath and Peter Langfelder[18]. It is a powerful and robust tool to analyse preselected gene sets and find correlated biological functions or related pathways to specific clinical traits. In our case, we used our MRS values of each metabolite as input for this analysis. The WGCNA tool is available on the bioconductor database and processed in R-software. In the following description we announce our used R-code.

Preparing Data for WGCNA

All expression data and values of each specific metabolite were used as input for WGCNA analysis. In addition, clinical data such as progression-free survival were included to expand the analysis. In a first step, outliers in the expression dataset were identified by a normalised connectivity below the threshold of -2.5. All samples identified as outliers were excluded from further analysis.

```
A=adjacency(t(datExpr),type="distance")
k=as.numeric(apply(A,2,sum))-1
Z.k=scale(k)
thresholdZ.k=-2.5
```

All analysis was performed as signed network analysis with a soft threshold (power ß) based on scale-free topology criteria. We tested different ß-values by following r-function.

```
powers=c(1:20)
sft=pickSoftThreshold(datExpr,powerVector=powers, networkType = "unsigned")
```

Calculation of SFT:

$$R_{SFT} = |cor(\log(freq_{KN}), \log(KN))| > 0.9$$

If the correlation between the logarithm of connectivity (KN) and frequency of connectivity ($freq_{KN}$) achieved values above 0.9, we assume that SFT criteria are adhered. The calculated power was implemented in the WGCNA analysis. In addition, dynamic tree cutting was used as described by Peter Langfelder[14] with a deep split of two.

```
blockwiseModules(datExpr,corType="pearson",
maxBlockSize=8000,networkType="signed",power=18,minModuleSize=30,
mergeCutHeight=mergingThresh, numericLabels=TRUE,
pamRespectsDendro=F,deepSplit=2)
```

Genes were divided into groups with its connected partner genes. All genes belonging to a cluster branch were defined as a “module”. These modules were used for all further analysis.

Characterising of Module Functions by Module Related Eigengenes

Eigengenes of all identified modules were calculated by following R-code and a cluster of cluster analysis was performed.

```
MEList=moduleEigengenes(datExpr,colors=moduleColorsAutomatic)
```

We calculated values for the module membership by module eigengene based connectivity (kME). This intramodule connectivity was correlated to each metabolite. Significantly positive or negative correlated modules were separated. We only used modules that were

significantly correlated ($p<0.01$). kME values of each module were used to characterize the biological functions or associated pathways.

```
datKME=signedKME(datExpr, ME_Merged)

colorOfColumn=substring(names(datKME),4)
```

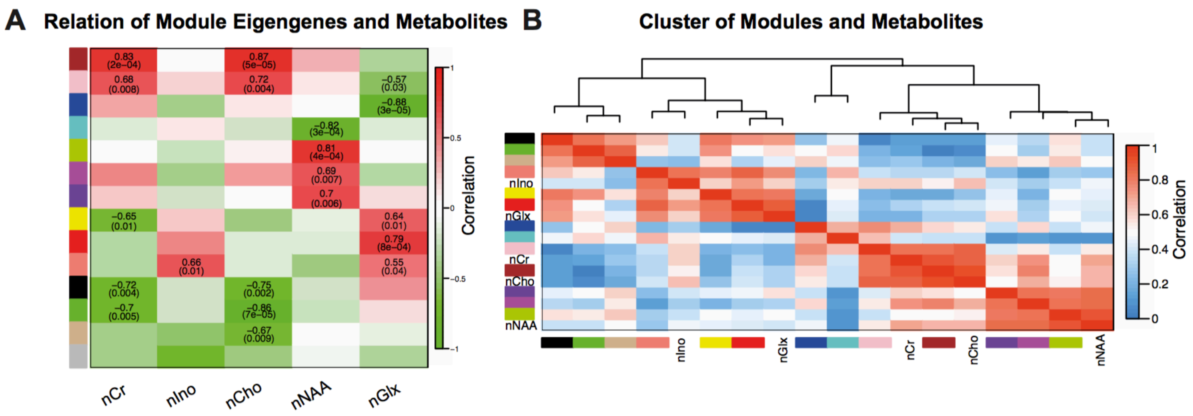

Supplementary Figure 1

A: An example of a heatmap by correlation values between module eigengenes and MRS metabolites. Positive correlations are marked in red, negative correlations in green.

B: Cluster of Clusters. Each clustered module and MRS metabolites were clustered by unsupervised Euclidean distance.

Gene Set Enrichment Analysis (GSEA)

Permutation based gene set enrichment analysis (GSEA)[17] was performed for each module to find specifically enriched biological functions and related pathways. Preranked GSEA were performed with 1000 permutations. The Molecular Signatures Database version 5.0 was used

including GO gene sets (C5) and pathway analysis gene sets (H1) (http://www.broadinstitute.org/gsea) as input databases for this analysis.
